# Effects of Ferulic Acid and *Angelica archangelica* Extract (Feru-guard^ ®^) on Mild Cognitive Impairment: A Multicenter, Randomized, Double-Blind, Placebo-Controlled Prospective Trial

**DOI:** 10.3233/ADR-200211

**Published:** 2020-09-18

**Authors:** Chiaki Kudoh, Tomokatsu Hori, Shunji Yasaki, Ryu Ubagai, Takeshi Tabira

**Affiliations:** a Kudoh Clinic for Neurosurgery & Neurology, Ota-ku, Tokyo, Japan; b Moriyama Neurological Center Hospital, Edogawa-ku, Tokyo, Japan; c Department of Neurology, Shin-Yurigaoka General Hospital, Furusawa, Aso-ku, Kawasaki, Japan; d Department of Diagnosis, Prevention and Treatment of Dementia, Graduate School of Medicine, Juntendo University, Bunkyo-ku, Tokyo, Japan

**Keywords:** *Angelica archangelica*, dementia, dietary supplement, double-blind randomized trial, ferulic acid, mild cognitive impairment

## Abstract

We conducted a multicenter, randomized, double-blind, placebo-controlled prospective trial examining a supplement containing ferulic acid and *Angelica archangelica* extract (Feru-guard^ ®^) for mild cognitive impairment (MCI). In the intention-to-treat population, Mini-Mental State Examination (MMSE) scores were significantly better at 24 weeks (*p* = 0.041) in the active group. In the per protocol population, MMSE was significantly better in the active group at 24 weeks (*p* = 0.008), and mixed effect models for repeated measures (MMRM) showed significant difference (*p* = 0.016). ADAS-Jcog was significantly better at 24 (*p* = 0.035) and 48 weeks (*p* = 0.015) in the active group, and MMRM was significant (*p* = 0.031). Thus, Feru-guard^ ®^ may be useful for MCI.

## INTRODUCTION

The concept of mild cognitive impairment (MCI) was proposed by Petersen [1], and several definitions of MCI have been established. The condition is characterized by the presence of cognitive impairment without impairment of instrumental or basic activities of daily living. Although physical and cognitive exercise and appropriate nutrition are currently recommended for MCI [2], the efficacy of these treatments is unsatisfactory. Thus, there are ongoing efforts to find better treatments, including functional foods and supplements [3].

Ferulic acid is a phytochemical found in the cell wall of plants. Recent studies have indicated that ferulic acid decreases free radicals, chronic inflammation, and beta-secretase transcription, resulting in reduction of amyloid-β (Aβ) and Aβ-induced neuronal loss [4– 7].

*Angelica* is a biennial plant from the umbelliferous family *Apiaceae.* Extracts from the root of *Angelica gigas* Nakai and the isolated coumarin-type chemical compounds decursin, decursinol, and decursinol angelate provide neuroprotective and cognitive enhancement effects under both *in vitro* and *in vivo* [8], and *A. archangelica* reduces the activity of acetylcholinesterase [9].

An open-label study demonstrated that a daily dose of Feru-guard^ ®^ 100 M (FG) ameliorated behavioral and psychiatric symptoms of dementia such as delusions, hallucinations, aggression, and anxiety in frontotemporal lobar degeneration and Lewy body dementia [10]. In another open-label study, participants with MCI showed slight cognitive improvement at 24, 48, and 96 weeks after oral supplementation with FG, although the change from baseline was not significant [11]. In the current study, we investigated the effects of FG on cognitive functioning in MCI individuals in a double-blind placebo-controlled trial.

## METHODS

Participants were recruited from the outpatient clinic of the Kudoh Clinic for Neurosurgery & Neurology, Shin-Yurigaoka General Hospital, Moriyama Neurological Center Hospital, Tokyo Clinic, and Ibara City Hospital, Ibara, Okayama, Japan. We enrolled participants aged 65 to 85 years old with MCI, according to the MCI criteria [1] and Mini-Mental State Examination (MMSE) scores ≥24, Alzheimer’s Disease Assessment Scale-Cognitive Subscale, Japanese version (ADAS-Jcog; 11 item, total score 70) scores 3– 10, Mild Cognitive Impairment Scale (MCIS) scores ≤49.8, Clinical Dementia Rating scores = 0.5, and 15-item Geriatric Depression Scale scores ≤10. For inclusion, participants had to meet these scoring criteria on two or more of the three scales MMSE, ADAS-Jcog, and MCIS. Excluded were those who had: 1) dementia, 2) neurodegenerative disorders; 3) medicine for cognitive impairment; 4) supplements affecting cognitive functions within 1 year before this study started; 5) history of depression and treatment; 6) participation in other medical prospective studies; 7) cognitive impairment owing to hypothyroidism, vitamin deficiency (B1, B6, B12, and folic acid), idiopathic normal pressure hydrocephalus, head injury, epilepsy, and encephalitis; 8) history of psychiatric disorders such as schizophrenia, alcoholism and drug addiction; 9) diabetes mellitus with HbA1c ≥8.0; 10) metabolic syndrome; 11) malignant neoplasm, acute inflammation, severe grade anemia, liver dysfunction, or renal dysfunction; and 12) judgment as ineligible for the trial by a responsible physician. Each participant or their family members provided written informed consent after receiving a detailed explanation of the study objectives and procedure.

### Supplement

The supplement containing ferulic acid and *A. archangelica* (Feru-guard^ ®^ 100 M; FG) and the placebo were provided by Glovia Co., Ltd. (Glovia, Tokyo, Japan). The FG daily dose contained 200 mg of ferulic acid and 40 mg of *A. archangelica* extract. The placebo daily dose contained 8.4 mg of stearic acid calcium and 271.6 mg of dextrin. Participants took the supplement or placebo before breakfast and dinner every day throughout the trial period.

Acute toxicity has been examined by outsourcing laboratories, and the LD50 was reported as >2 g/kg for ferulic acid and >600 mg/kg for *A. archangelica* extracts in rodents. Ferulic acid and *A. archangelica* extracts are extracted and refined from rice bran and *A. archangelica* roots, respectively, and the quality is strictly controlled by the production companies. In Japan, so far over 50,000 people have taken over 1.2 million boxes of Feru-guard^ ®^, but no serious adverse events have been reported.

### Study design

This was a multicenter, randomized, double-blind, placebo-controlled, prospective trial designed and conducted by trial committee members. Participant assignment was performed using the cloud dynamic assignment minimization method. The collected data were entered into an online system by an employee of Glovia. As the data were blinded, the person performing the input was not able to assess the data. All data were strictly managed using an internet system with a security code, and could not be viewed unless the security key was opened.

The study protocol adhered to the Declaration of Helsinki (2013) and the Ethical Guidelines for Medical and Health Research Involving Human Subjects. The study was approved by the ethical committee of Mizuo-Clinic (approval No. 160427) and was registered in the UMIN Clinical Trials Registry (ID: UMIN000024063). The ethical committees of each participating hospital also approved the study protocol.

In the protocol, 200 participants were scheduled to participate in the study from September 1, 2016, to June 30, 2021, and the study period was 2 years after entry. However, because a new clinical trials act was announced in March 2019 by the Japanese Ministry of Health, Labour and Welfare, it was necessary to terminate the study on December 31, 2018. Therefore, the study period was limited to 48 weeks, and the final number of participants enrolled in the study was 56.

### Clinical assessments

ADAS-Jcog, MMSE, and MCIS assessment were conducted at baseline, 24 weeks, and 48 weeks. Physical examinations and laboratory tests including blood examination measurements, electrocardiography, and magnetic resonance imaging were conducted at baseline. Blood tests were performed on the complete blood count, blood urea nitrogen, creatinine, total bilirubin, aspartate aminotransferase, alanine aminotransferase, *γ*-glutamyltranspeptidase, alkaline phosphatase, low density lipoprotein cholesterol, high sensitivity C-reactive protein, hemoglobin A1c, thyroid stimulating hormone, and free tetraiodothyronine.

The primary outcome measure to assess the effect of FG on cognitive functions was ADAS-Jcog score, and the secondary outcome was MMSE score at 24 and 48 weeks, respectively. Assessment of each score in the apolipoprotein E (ApoE 4^ +^ and ApoE4^–^) groups was also included in the secondary outcome. ApoE genotypes were determined by polyacrylamide gel isoelectric focusing [12] using blood samples collected at baseline.

### Statistical analysis

Statistical analysis was conducted using Mann-Whitney *U* tests or Wilcoxon signed-rank tests. Changes in the average scores for 24 to 48 weeks were compared between the two groups using a mixed effect model for repeated measures (MMRM). All statistical analyses were performed by an outsourcing company (Kureha Special Laboratory Co., Tokyo) using SAS version 9.4 (SAS Institute Inc., Cary, NC, USA). Two-tailed tests were used and significance was set at *p* <  0.05.

## RESULTS

**Fig. 1:**
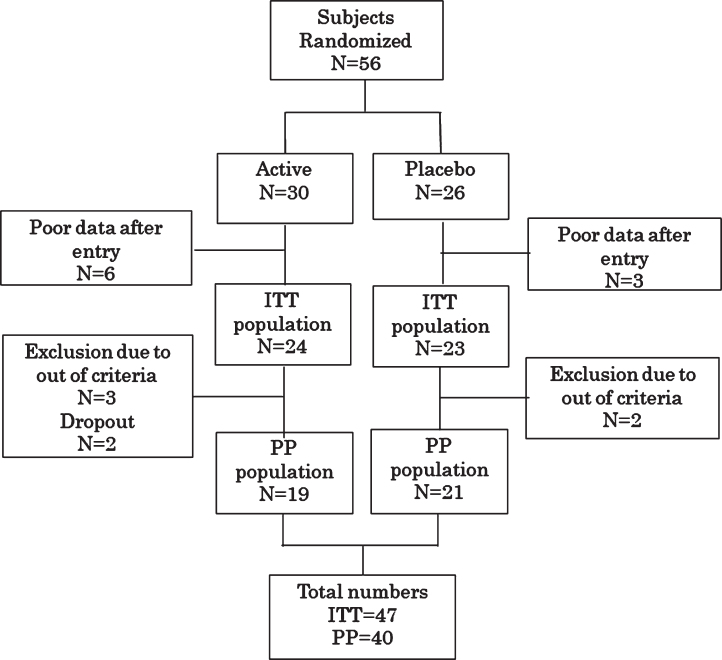
Randomization and assignment of study subjects. Please see the text for reasons for exclusion. ITT, intention-to-treat; PP, per protocol.

In total, 56 individuals were randomly allocated. Nine individuals (5 active and 4 placebo) were excluded as they had insufficient follow-up data. Therefore, 47 cases were examined as the intention-to-treat (ITT) population. Furthermore, 7 individuals were excluded from the ITT population before the data key was opened; 3 were found not to meet the inclusion criteria, 1 was judged as normal, and 1 was found to have already taken FG before the start of the study. In addition, 2 cases were excluded as dropouts owing to poor adherence. Therefore, data for 40 per protocol (PP) individuals were analyzed (Fig. 1). Baseline characteristics of the ITT subjects are summarized in Table 1.

**Table 1:** Baseline characteristics of the intention-to-treat population

		Active (*n* = 24)	Placebo (*n* = 23)	*p*
Age	Median [min– max]	75.0 [67.0– 85.0]	78.0 [69.0– 84.0]	0.1175
Sex	Male	11 (45.8%)	8 (34.8%)	0.5556
Female	13 (54.2%)	15 (65.2%)
Apo E	*ɛ*2/3	2 (8.7%)	0 (0.0%)	0.2176
	*ɛ*3/2	0 (0.0%)	1 (4.8%)
	*ɛ*3/3	13 (56.5%)	17 (81.0%)
	*ɛ*3/4	4 (17.4%)	1 (4.8%)
	E4/3	2 (8.7%)	2 (9.5%)
	*ɛ*4/4	2 (8.7%)	0 (0.0%)
	Not specified	1	2
Apo E	*ɛ*4	8 (34.8%)	3 (14.3%)	0.1685
	Non *ɛ*4	15 (65.2%)	18 (85.7%)
	Not specified	1	2
Baseline ADAS-Jcog	Median (N) [min– max]	6.7 (*n* = 23) [2.7– 12.0]	7.6 (*n* = 23) [4.0– 18.3]	0.1798
Baseline MCIS	Median (N) [min– max]	44.1 (*n* = 24) [15.4– 61.2]	39.3 (*n* = 23) [20.9– 63.0]	0.1906
Baseline MMSE	Median (N) [min– max]	27.0 (*n* = 24) [21.0– 29.0]	26.0 (*n* = 21) [22.0– 30.0]	0.2803

This table shows the baseline characteristics of the intention-to-treat groups. Differences between the active and placebo groups were analyzed using the Mann-Whitney’s *U* test for continuous variables and Fisher’s exact test for discrete variables. ApoE, apolipoprotein E; MMSE, Mini-Mental State Examination; ADAS-Jcog, Alzheimer’s Disease Assessment Scale-Cognitive Subscale, Japanese version; MCIS, Mild Cognitive Impairment Scale.

**Fig. 2:**
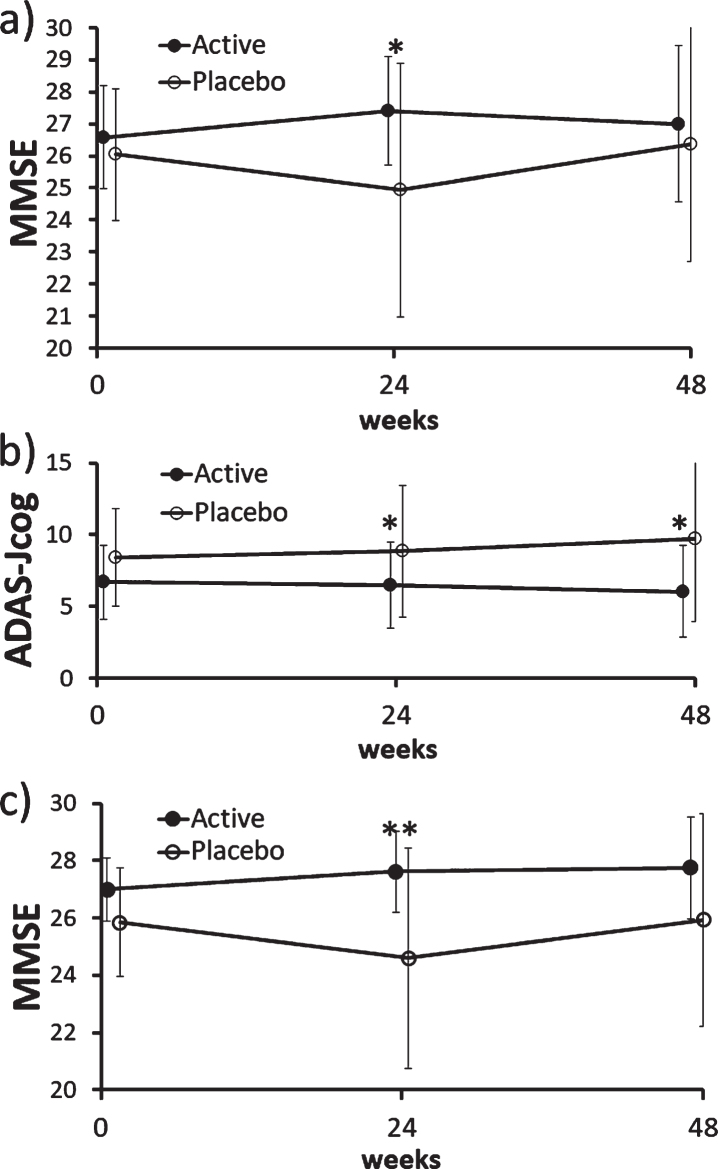
Changes in MMSE and ADAS-Jcog scores in the ITT and PP population. a) There was a significant difference in MMSE scores between the active (FG) and placebo groups at 24 weeks in the ITT population (*p* = 0.041; Mann– Whitney *U* test). b) There was a significant difference in ADAS-Jcog scores between the active (FG) and placebo groups at 24 weeks (*p* = 0.035) and 48 weeks (*p* = 0.015; Mann– Whitney *U* test) in the PP population. The MMRM analysis also showed a significant between-group difference (*p* = 0.031). c) There was a significant difference in MMSE scores between the active (FG) and placebo groups at 24 weeks (*p* = 0.008; Mann– Whitney *U* test) and the mixed effect model for repeated measures also showed a significant difference (*p* = 0.016) in the PP population. ADAS-Jcog, Alzheimer’s Disease Assessment Scale-Cognitive Subscale, Japanese version; ITT, intention-to-treat; MMSE, Mini-Mental State Examination; MMRM, mixed effect model for repeated measures, PP, per protocol.

There was no difference in ADAS-Jcog, MMSE, and MCIS scores between the active (FG) and placebo groups at baseline in the ITT population (Table 1), indicating adequate randomization. In the ITT population, there was no between-group difference in ADAS-Jcog scores (primary endpoint) including its item scores, but MMSE scores (secondary endpoint) were significantly better in the active group than placebo (*p* = 0.041) at 24 weeks (Fig. 2a).

In the analysis of PP population, MCIS and word recognition of ADAS-Jcog were significantly better (*p* = 0.048 and *p* = 0.04, respectively) in placebo at baseline, but the follow-up data and the MMRM analysis were not significant.

ADAS-Jcog scores were significantly better at 24 (*p* = 0.035) and 48 weeks (*p* = 0.015) in the active group than placebo of the PP population (Fig. 2b), where MMRM showed significant between-group difference in changes in average scores at 24 and 48 weeks (*p* = 0.031).

In the sub-analysis of each item of ADAS-Jcog, word recall was significantly better in the active group at 24 weeks (*p* = 0.048), and orientation was significantly improved in the active group at 48 weeks (*p* = 0.026). Otherwise, there was no statistical difference in other items.

MMSE scores were also better in the active group at 24 weeks in the PP population (*p* = 0.008), and MMRM also showed significant difference (*p* = 0.016) (Fig. 2c).

Cluster analyses between ApoE4+ and ApoE4– groups were all negative. Cluster analyses using MMSE scores, ADAS-Jcog scores, and age at baseline also showed no significant differences. Clinical and laboratory observations showed no side effects.

## DISCUSSION

Although MCIS and word recognition of ADAS-Jcog showed a statistical difference in the baseline of the PP population, the difference was not significant at 24 and 48 weeks, and the MMRM showed no significant differences. Therefore, we believe that these findings do not undermine the superiority of FG.

Ferulic acid directly destabilizes Aβ fibrils and inhibits Aβ aggregation [13, 14]. Interaction of Aβ with ferulic acid occurs during the initial stage of the aggregation process, and disruption of the peptide self-assembly results in redirection of non-fibrillar amorphous aggregate formation [15]. However, its bioavailability remains to be elucidated.

One of the effective molecules in *Angelica* is decursin. In addition to its antioxidant and anti-inflammatory effect, it was also found to lower calcium influx in glutamate-treated rat cortical cells [16]. Moreover, decursin greatly inhibited apoptosis by suppressing caspase-3 activity in Aβ _25–35_ treated PC12 cells [8]. Furthermore, decursin decreased the mitochondrial membrane potential, which inhibited production of reactive oxygen species, and reduced the output of cytochrome c [17]. The neuroprotective effects of decursinol were higher against kainic acid-induced neurotoxicity [16], and it showed a significant inhibition of acetylcholinesterase [18]. Thus, ferulic acid and *Angelica* extract appear to reduce various pathological mechanisms of AD.

It would be interesting to investigate whether FG can delay conversion from MCI to AD. The above-mentioned study of the effects of FG on MCI suggested an annual conversion rate of approximately 8.35% [11]. It was 18.3% in the Alzheimer’s Disease Neuroimaging Initiative (ADNI) [19], and was 28.8% in the Japanese ADNI [20]. Thus, FG may delay the conversion from MCI to dementia. Further studies are required to confirm this.

One of the limitations of this study is that we did not discriminate between amnestic and non-amnestic MCI, or between AD-type and non-AD-type MCI. We plan to do this in future studies.

In conclusion, the current study demonstrated the clinical effectiveness of ferulic acid and *A. archangelica* extract on cognitive functioning among older adult individuals with MCI.

## CONFLICT OF INTEREST

Kudoh Clinic for Neurosurgery & Neurology, Moriyama Neurological Center Hospital, and Ibara City Hospital received a research grant from Glovia. Glovia paid a contract fee to a temporary agency for a temporary employee unrelated to Glovia, whose services were accepted by TH. Glovia provided the supplement for the study.
